# Comment on “Analysis and Thoughts about the Negative Results of International Clinical Trials on Acupuncture”

**DOI:** 10.1155/2015/637215

**Published:** 2015-11-24

**Authors:** Leigh Jackson

**Affiliations:** Private Practice, 72 Ashdown Way, London SW17 7TH, UK

According to Liu et al. [1] acupuncture studies showing negative results, when conducted outside China, must be wrong. Apparently, acupuncture trials rarely, if ever, produce negative results in China because acupuncture is always performed correctly by experts. Is this true or could it be the case that bias is endemic in clinical trials of acupuncture in China?

Liu et al. criticise several trials because acupuncture was not used appropriately according to traditional practice. However, all treatments being offered to patients should be subject to the highest standards of clinical testing whether they conform to traditional practice or are innovative in nature.

It is nearly two decades since the WHO asked for acupuncture to be tested by the best clinical methods and the world is still waiting for sound evidence to show that acupuncture is superior to placebo. Whilst there is an enormous quantity of poor quality positive studies there are now many high quality negative studies.

*Some Comments on Liu et al.'s Higher Rated Trials*. Effective blinding and appropriate controls are essential elements of a good clinical trial. Only one study was rated by Liu et al. with a Jadad top score of 5: Park et al. [2]. This moxibustion study for constipation had a sham control, unlike previous studies judged by Park et al. to have a high risk of bias. No difference was found between acupuncture and sham by Park et al.

In contrast, Smith et al. [3], rated a Jadad score of 4 by Liu et al., only had a waiting list control. Any form of therapeutic support for women experiencing stress due to fertility problems can be expected to be warmly welcomed by patients and the study shows that was very much the case. The intention of acupuncture was to help with women's psychological ability to cope with the stress of their fertility problems. It would be astonishing if patients receiving some kind of support did not find that more helpful in dealing with their stressful situation, than being stuck on a waiting list. Blinding was minimal. In no way could this trial ever establish any benefit to patients over and above placebo effect. This trial guaranteed the result which it obtained.

Comparing the results of these two studies, whilst one result was entirely to be expected the other was unpredictable. One result was physical and the other psychological. Park et al.'s rationale was a doubt concerning the safety of previous trials. Liu et al. may say that moxibustion was an inappropriate treatment. That is not the fault of Park et al. who were justified in undertaking a new study with superior methodology to test dubious results of previous studies.

These two contrasting RCTs show that what is important is not a general comparison of overall rates of quality of blinding across positive and negative studies. Indeed, that comparison can be misleading. What matters is the quality and relevance of each individual study. In other words, each trial must be judged on its own merits. The minimal blinding in Smith et al. was irrelevant since the result was a foregone conclusion. The absence of a sham control was the most severe failing of this study. Double blinding and sham control in Park et al. meant the result was not a foregone conclusion.

Other positive trials were given a high Jadad rating: Paterson et al. [4]; Shiflett and Schwartz [5]; Liodden et al. [6].

*These Studies All Have Serious Problems*. Paterson et al.'s trial does not qualify for a Jadad score of 4 because it was not a double blind trial. It was an open trial exposed to high risk of bias.

Shiflett and Schwartz reanalysed one trial out of a set of three, designed to assess the value of acupuncture and amitriptyline for pain relief from peripheral neuropathy in HIV males. The set of trials was negative. Employing different statistical methodology to the original trials, Shiflett and Schwartz found a nominal statistical difference for acupuncture (*P* = 0.047) though it was not for pain relief. The rationale for this reanalysis was to see if it confirmed another reanalysis using their methodology, on another trial from the set, in which acupuncture and amitriptyline were both found to be more effective than placebo for pain relief, contradicting the findings of the original trial. This means that the two reanalyses of the two earlier studies, using a different methodology to the original trials, which produced consistent results across the set of trials, themselves produced contradictory results. The relevance of their results is questionable.

Liodden et al.'s trial does not qualify for a Jadad score of 4. It was an open trial, whose authors acknowledge was wide open to bias.

Two trials employed commendable methodology: Modlock et al. [7] and Pastore et al. [8].

They both found acupuncture to fare no better than placebo.

On the question of the validity of placebo forms of acupuncture, if acupuncture is so variably defined as to render a valid placebo form of acupuncture quite impossible, then acupuncture cannot claim to be more than a placebo on any other basis than pure faith.

In relation to the vast variability of acupuncture, another trial included in Liu et al. is instructive: Rogha et al. [9]. This was a double blind placebo controlled trial which concluded that acupuncture offered short term benefit for tinnitus. The study employed traditional individualised treatment. Different acupoints were employed for different patients. Coupled with the use of nonpuncturing sham needles the results could imply that needle placement is not important. It is also possible that blinding failed as there is no mention of its effectiveness.

On the evidence presented in the study by Liu et al. acupuncture trials worldwide are routinely exposed to high risk of bias, knowingly or otherwise. Good methodology results in acupuncture failing to outperform placebo.

## References

[1] Liu W.-H., Hao Y., Han Y.-J., Wang X.-H., Li C., Liu W.-N. (2015). Analysis and thoughts about the negative results of international clinical trials on acupuncture. *Evidence-Based Complementary and Alternative Medicine*.

[2] Park J.-E., Sul J.-U., Kang K., Shin B.-C., Hong K.-E., Choi S.-M. (2011). The effectiveness of moxibustion for the treatment of functional constipation: a randomized, sham-controlled, patient blinded, pilot clinical trial. *BMC Complementary and Alternative Medicine*.

[3] Smith C. A., Ussher J. M., Perz J., Carmady B., De Lacey S. (2011). The effect of acupuncture on psychosocial outcomes for women experiencing infertility: a pilot randomized controlled trial. *Journal of Alternative and Complementary Medicine*.

[4] Paterson C., Taylor R. S., Griffiths P. (2011). Acupuncture for ‘frequent attenders’ with medically unexplained symptoms: a randomised controlled trial (CACTUS study). *The British Journal of General Practice*.

[5] Shiflett S. C., Schwartz G. E. (2011). Effects of acupuncture in reducing attrition and mortality in HIV-infected men with peripheral neuropathy. *Explore*.

[6] Liodden I., Howley M., Grimsgaard A. S. (2011). Perioperative acupuncture and postoperative acupressure can prevent postoperative vomiting following paediatric tonsillectomy or adenoidectomy: a pragmatic randomised controlled trial. *Acupuncture in Medicine*.

[7] Modlock J., Nielsen B. B., Uldbjerg N. (2010). Acupuncture for the induction of labour: a double-blind randomised controlled study. *BJOG: An International Journal of Obstetrics & Gynaecology*.

[8] Pastore L. M., Williams C. D., Jenkins J., Patrie J. T. (2011). True and sham acupuncture produced similar frequency of ovulation and improved LH to FSH ratios in women with polycystic ovary syndrome. *The Journal of Clinical Endocrinology and Metabolism*.

[9] Rogha M., Rezvani M., Khodami A. R. (2011). The effects of acupuncture on the inner ear originated tinnitus. *Journal of Research in Medical Sciences*.

